# Mitochondrial alternative oxidase contributes to successful tardigrade anhydrobiosis

**DOI:** 10.1186/s12983-021-00400-5

**Published:** 2021-04-01

**Authors:** Daria Wojciechowska, Andonis Karachitos, Milena Roszkowska, Wiktor Rzeźniczak, Robert Sobkowiak, Łukasz Kaczmarek, Jakub Z. Kosicki, Hanna Kmita

**Affiliations:** 1 Department of Macromolecular Physics, Faculty of Physics, Adam Mickiewicz University, Poznań, Poland; 2 Department of Bioenergetics, Institute of Molecular Biology and Biotechnology, Faculty of Biology, Adam Mickiewicz University, Poznań, Poland; 3 Department of Animal Taxonomy and Ecology, Institute of Environmental Biology, Faculty of Biology, Adam Mickiewicz University, Poznań, Poland; 4 Department of Cell Biology, Institute of Experimental Biology, Faculty of Biology, Adam Mickiewicz University, Poznań, Poland; 5 Department of Avian Biology and Ecology, Institute of Experimental Biology, Faculty of Biology, Adam Mickiewicz University, Poznań, Poland

**Keywords:** Anhydrobiosis, Mitochondrial alternative oxidase, Tardigrade, *Milnesium inceptum*

## Abstract

Anhydrobiosis can be described as an adaptation to lack of water that enables some organisms, including tardigrades, to survive extreme conditions, even some that do not exist on Earth. The cellular mechanisms underlying anhydrobiosis are still not completely explained including the putative contribution of mitochondrial proteins. Since mitochondrial alternative oxidase (AOX), described as a drought response element in plants, was recently proposed for various invertebrates including tardigrades, we investigated whether AOX is involved in successful anhydrobiosis of tardigrades. *Milnesium inceptum* was used as a model for the study. We confirmed functionality of *M. inceptum* AOX and estimated its contribution to the tardigrade revival after anhydrobiosis of different durations. We observed that AOX activity was particularly important for *M. inceptum* revival after the long-term tun stage but did not affect the rehydration stage specifically. The results may contribute to our understanding and then application of anhydrobiosis underlying mechanisms.

**Supplementary Information:**

The online version contains supplementary material available at 10.1186/s12983-021-00400-5.

## Introduction

Water availability is one of the most important factors for life. However, terrestrial habitats may endure occasional lack of water that requires specific adaptations from inhabiting organisms at different levels of biological organization. One of the most prevalent adaptations is anhydrobiosis, often called simply “life without water” [1–4]. The phenomenon is also known as desiccation tolerance, defined as the ability to dry to equilibrium with moderately to very dry air (i.e., to 10% water content or even less) and then recover to normal functioning after rehydration without sustaining damage [5]. A full explanation of the mechanisms underlying anhydrobiosis and identification of biomarkers of successful anhydrobiosis may benefit different applications that rely on organism tolerance to extreme environmental conditions. Such applications could include, for example, dry vaccines, preservation of biological materials for transplantation or food production, enzymes working in a small amount of water and mechanisms of DNA protection and repair [4, 6–8].

Anhydrobiosis occurs in many microorganisms as well as in plants and some small invertebrates, and among the latter the best-known examples are tardigrades [4, 5, 9–15]. Tardigrade anhydrobiosis includes entering, permanent and leaving stages, which correspond to the dehydration (i.e., tun formation), tun and rehydration stages, respectively (e.g. [12]). In the tun stage a tardigrade is dehydrated and shrank to about 30% of its original volume, taking on a shape resembling a barrel via contraction of the anterior-posterior body axis, retraction of the legs and rearrangement of internal organs and cells. These changes are reversed during the rehydration stage [2, 10, 12, 13, 16]. Thus, on the organismal level, anhydrobiosis in tardigrades, is fairly well understood.

However, relevant molecular mechanisms are still not fully explained. Searching on the mechanisms has revealed several common components in anhydrobiotic animals. Accordingly, LEA proteins and heat shock proteins have been shown to be important for these animals’ successful anhydrobiosis while other mechanisms are more prominent in some of these animals than others. For example *Artemia* shrimps and nematodes produce significant levels of trehalose to replace water as cells dehydrate, while some species of tardigrades produce none at all [2, 9, 17, 18]. On the other hand, three families of intrinsically disordered proteins were identified only in tardigrades, namely the Cytoplasmic-, Secreted-, and Mitochondrial- Abundant Heat Soluble (CAHS, SAHS, and MAHS, respectively) proteins, collectively termed Tardigrade Disordered Proteins (TDPs). These proteins are highly expressed constitutively or significantly enriched in response to desiccation (e.g. [18, 19],) but seem to be missing in some tardigrades, suggesting that anhydrobiosis in tardigrades cannot be attributed solely to TDPs [20, 21]. However, available data appear to support a common and important role of proper mitochondria function in anhydrobiosis. Accordingly, it has been shown that mitochondria uncoupling abolishes tun formation [22], and the organelles contribute to tun functionality and successful rehydration [23–25], but their role in tardigrade anhydrobiosis still remains elusive. Therefore, studies of mitochondrial proteins that are involved in surviving stress conditions should contribute to our understanding of the role of mitochondria in anhydrobiosis.

One of the mitochondrial proteins important in the response to drought stress is alternative oxidase (AOX) [26–29], the mitochondrial inner membrane, cyanide-insensitive, iron-binding protein that introduces a branch into the mitochondrial respiratory chain (MRC) at the coenzyme Q level. This allows for electron transfer from MRC complexes I and II via coenzyme Q to oxygen without involvement of the MRC cytochrome pathway composed of complexes III and IV [26, 29, 30]. As a result, AOX provides a bypath that releases constraints on the MRC cytochrome pathway and consequently participates in mitochondrial reduction-oxidation reactions important for cell metabolic plasticity involved in adaptation to variable biotic and abiotic stress factors. Accordingly, heterologous expression of AOX in cultured mammalian cells, fruit flies and mice under mitochondrial stress conditions restores respiratory activity and corrects metabolism (e.g. [31]).

AOX was initially considered to be limited to plants as well as some fungi and protists but its presence has recently been proposed in different invertebrate animals, except for insects [28, 29, 31–33]. Analysis of available but scarce tardigrade genomic and/or transcriptomic data indicates the presence of AOX encoding gene in three tardigrade species [21, 28] including *Milnesium inceptum* [34] (formerly named *Milnesium tardigradum*), known to be highly resistant to periodical dehydration under laboratory conditions (e.g., [17]). Thus, in this study we tested the hypothesis that AOX is involved in tardigrade anhydrobiosis using *M. inceptum* as a model for the research. To test this hypothesis, we verified the functionality of *M. inceptum* AOX and then estimated animals’ recovery to full activity after dehydration or rehydration in the presence of an AOX inhibitor and after different durations of the tun stage. The results indicate that AOX activity is important for tun revival, as reflected by tardigrades’ ability to return to full activity, but does not affect the rehydration stage itself. Additionally, the contribution of AOX depends on the duration of the tun stage.

## Materials and methods

### Detection of heterologously expressed *M. inceptum* AOX activity in mitochondria of intact *Saccharomyces cerevisiae* cells

For heterologous expression of *M. inceptum* AOX, the coding sequence of the protein was identified by bioinformatics methods (for details see electronic supplementary material, Figure S1 and Figure S2). The expression was performed by CRISPR/Cas9 and *S. cerevisiae* BY4741 strain (*MATa, his3Δ, leu2Δ, met15Δ, ura3Δ*) (EUROSCARF) transformation by electroporation (for details see electronic supplementary material, Figure S3). Cells of the resulting BY4741 + AOX yeast strain (*MAT*a, *his3*Δ, *leu2*Δ, *met15*Δ, *ura3*Δ, *gal1*Δ::*Mi-AOX*) were grown in YPG medium (1% yeast extract, 2% peptone, 3% glycerol, pH 5.5) containing a non-fermentable carbon source that requires functioning mitochondria [35]. The cells were grown to OD_550_ = 1, then galactose at a final concentration of 2% was added to induce and maintain AOX expression for 24 h. In parallel, control yeast cells were cultured in the absence of galactose. Then, yeast cells were washed twice with double distilled water (ddH_2_O), resuspended in 100 μL of YPG and quantified by OD_550_ measurement. For the same amount of cells, rates of oxygen uptake were determined in 1 mL of YPG using a Clark electrode (Hansatech Instruments). To determine bioenergetics parameters related to AOX activity, 1 mM KCN (to inhibit the MRC complex IV, i.e., cytochrome c oxidase) and 3 mM BHAM (to inhibit alternative oxidase) were applied. The applied final concentrations of KCN and BHAM were verified experimentally to achieve saturation.

### Detection of *M. inceptum* AOX presence in isolated *S. cerevisiae* mitochondria

Mitochondria of *S. cerevisiae* cells expressing AOX were isolated according to the standard procedure [36]. Protein concentration was measured by the method of Bradford and albumin bovine serum (BSA), essentially fatty acid free, was used as a standard. The mitochondrial proteins were separated by SDS-PAGE in the presence of 6 M urea and gels were stained with Coomassie Brilliant Blue G-250.

### Culture of *M. inceptum*

The initial specimens of *M. inceptum* were collected in a xerothermic habitat: mosses on a concrete wall in the city centre of Poznań, Poland (52°24′15″N, 16°53′18″E, 87 m asl). The extraction was performed by the standard method [37]. To maintain the culture, *M. inceptum* specimens were kept at 18 °C in the darkness and at relative humidity (RH) of 40% (POL EKO KK 115 TOP+ climatic chamber) in covered Petri dishes (5.5 cm in diameter) with bottom scratched by sandpaper to allow tardigrade movements. Animals were coated with a thin layer of the culture medium being spring water (Żywiec Zdrój; Żywiec Zdrój S.A., Poland) mixed with ddH_2_O in 1:3 ratio. The culture medium was exchanged every week and the animals were fed with the nematode *Caenorhabditis elegans* Bristol N2 strain, and the rotifer *Lecane inermis* 1.A2.15 strain. The former was obtained from the Caenorhabditis Genetics Center at the University of Minnesota (Duluth, USA) and its culture was maintained by standard methods [38].

### *M. inceptum* anhydrobiosis protocol

For tun formation, fully active (displaying coordinated movements of the body and legs) *M. inceptum* adult specimens of medium body length (approximately 500–550 μm) were extracted from the culture. After removal of debris, the animals were transferred to 3.5 cm (in diameter) covered and vented Petri dishes with bottom scratched by sandpaper. In each dish, 10 specimens were placed in 400 μl of the culture medium and then dehydrated. Each of the experimental groups included four to six dishes. This denotes, for a total of 40–60 specimens per experimental group (Table 1). The dishes were allowed to dry slowly in the Q-Cell incubator (40–50% RH, 20 °C, darkness) for 72 h. Tun formation was checked once per 24 h period by a short observation (about 1 min) under an Olympus SZ61 stereomicroscope connected to an Olympus UC30 microscope digital camera. The dehydrated animals (tuns) were kept under the above conditions for 3, 30 or 60 days.

**Table 1:** Experimental groups applied in the studies. They included two different concentrations of BHAM during *M. inceptum* dehydration and rehydration, and one concentration of MitoTEMPO during *M. inceptum* dehydration (including proper controls) as well as different durations of the tun stage. The tested conditions are marked with the x

TUN DURATION (days)	EXPERIMENTAL GROUPS							
	DEHYDRATION					REHYDRATION		
	control 1 (0.3% v/v methanol)	0.1 mM BHAM	0.2 mM BHAM	control 2 (culture medium)	0.01 mM MitoTEMPO	control 1 (0.3% v/v methanol)	0.1 mM BHAM	0.2 mM BHAM
3	x	x	x	x	x	x	x	x
30	x	x	x					
60	x	x	x	x	x	x	x	x

After 3, 30 or 60 days of the tun stage duration, the tun rehydration was performed by addition of 3 ml of the culture medium to each dish, and the tuns were transferred to small glass cubes and kept at room conditions (18 to 20 °C, 40–50% RH and light conditions regulated by seasonal changes in day/night cycle; it should be mentioned that according to our observations, photoperiod does not affect return of *M. inceptum* tuns to active life). The animals’ return to full activity was observed in the glass cubes at chosen time points (i.e., time windows: 10, 20, 30, 40, 60, 90, 120, 180, 240, 360, 480 and 1440 min) for approximately 1 min under the stereomicroscope. The average number of specimens that recovered to full activity after 24 h (1440 min) from the onset of rehydration was defined as the final return to full activity.

BHAM, a known AOX inhibitor (prepared in methanol), and MitoTEMPO, a known mitochondria-specific superoxide scavenger (prepared in ddH_2_O), were added to the culture medium at the beginning of the dehydration or rehydration stage. The applied final concentrations of BHAM (0.1 and 0.2 mM) and MitoTEMPO (0.01 mM) were verified experimentally to avoid lethal effect. For that, we analysed animal mobility after addition of the compounds to the culture medium containing active animals. We assumed the absence of a lethal effect when approximately 90% of the treated animals moved their legs and body in a coordinated way 24 h after addition of the compounds. Because two different solvents were applied for BHAM and MitoTEMPO, two different controls were used in experiments: control 1 (C_1_) included animals dehydrated or rehydrated in the culture medium in the presence of suitably diluted methanol solution (0.3% v/v), and control 2 (C_2_) included animals dehydrated in the culture medium. To test for differences in the animals’ return to full activity, we compared the animals’ recovery at the applied time windows (10, 20, 30, 40, 60, 90, 120, 180, 240, 360, 480 and 1440 min) between experimental groups (Table 1) and for different time spent in the tun stage. For animals treated with BHAM, during both dehydration and rehydration, we distinguished three experimental groups (C_1_, 0.1 mM BHAM and 0.2 mM BHAM) and for animals treated with MitoTEMPO during dehydration, we distinguished two experimental groups (C_2_ and 0.01 mM MitoTEMPO).

### Chemicals

Manufacturers of the chemicals used are listed in electronic supplementary material, Table S1.

### Statistical analysis

Effects of BHAM and KCN on the rate of oxygen uptake displayed by *S. cerevisiae* cells were analysed by *t*-test.

For testing differences in number of tuns formed under different conditions (in the presence of MitoTEMPO or BHAM as well as for proper controls) one-way ANOVA [39] was applied.

To analyse the effect of BHAM presence during tun formation or rehydration and of MitoTEMPO presence during tun formation on anhydrobiotic animals’ recovery to full activity after different duration of the tun stage, Factorial ANOVA [39] was applied. For this analysis we used two grouping independent variables: (I) time windows of animal observations following rehydration and (II) the applied compound concentrations. The latter denotes two different concentrations of BHAM versus control conditions and one concentration of MitoTEMPO versus control conditions, where control conditions denote the absence of BHAM or MitoTEMPO. In this way we distinguished three experimental groups for testing of BHAM presence (C_1_, 0.1 mM BHAM and 0.2 mM BHAM) or two experimental groups for testing of the MitoTEMPO (C_2_ and 0.01 mM MitoTEMPO) (Table 1). Thus, differences in animals’ return to full activity were tested between the mentioned two grouping independent variables: (I) 12 time windows (10, 20, 30, 40, 60, 90, 120, 180, 240, 360, 480 and 1440 min) and (II) three experimental groups distinguished for BHAM (C_1_, 0.1 mM BHAM and 0.2 mM BHAM) or (III) two experimental groups distinguished for MitoTEMPO (C_2_ and 0.01 mM MitoTEMPO). Because of the application of the two grouping variables, it was impossible to simply test the differences between each group, as the effect of multiple comparisons would decrease the biological value of the results [40]. We used the standard presentation of results for Factorial ANOVA analysis [39, 40]. For each model we calculated *F*-statistics and R^2^ as a measure of variance explained [40]. For ANOVA models, when *F* was statistically significant (i.e., α-probabilities (*p*) < 0.05), in the next step we deepened that analysis by determination of statistically significant factors in the model (i.e., time window or experimental groups). For this purpose we performed *t*-test for each independent variable (electronic supplementary material, Table S2 and Table S3). If this test for the factor ‘experimental groups’ was statistically significant, it provided (from a methodical point of view) an authorization to perform Tukey post-hoc test [39]. Then the post-hoc test results were visualized in figures.

To compare effects of BHAM and MitoTEMPO on animals’ return to full activity, we analysed the ratios of average numbers of fully active animals obtained for BHAM- or MitoTEMPO-treated tardigrades to average numbers of fully active appropriate control animals for different duration of the tun stage. The analysis was performed, regardless of the time windows, using developed Linear Mixed Models for the duration of the tun stage (3 and 60 days) and the applied concentrations of BHAM (0.1 mM and 0.2 mM) and MitoTEMPO (0.01 mM). This procedure was justified by our observation that revival time was influenced by the time window (see Results). To control for that factor we developed models with time window as a random factor and concentration as a fixed effect. All calculations were performed using R [41].

## Results

### The predicted *M. inceptum* AOX is a functional protein

**Fig. 1:**
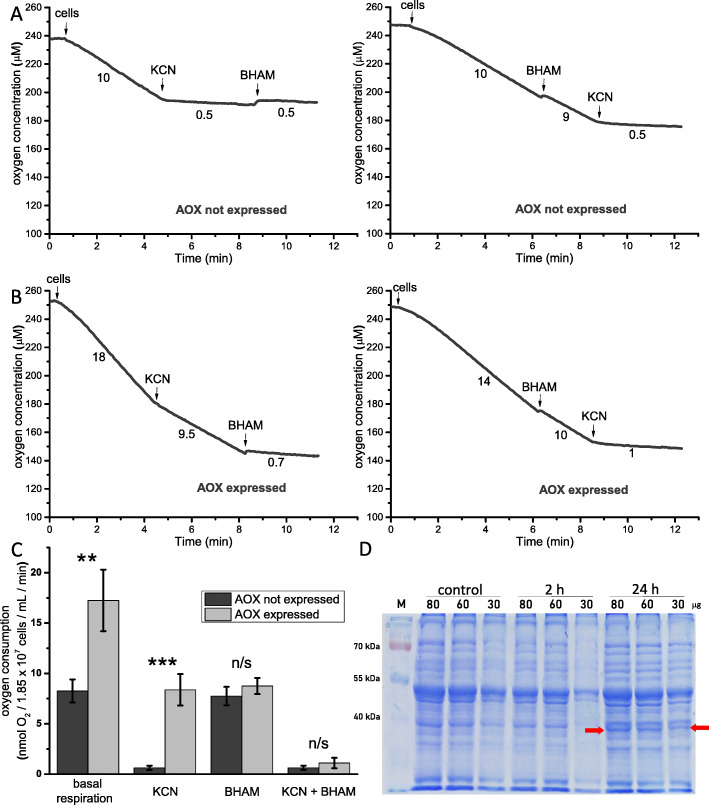
Functional analysis of *M. inceptum* AOX expressed in *S. cerevisiae* mitochondria. Tardigrade AOX expression was induced by the addition of galactose to YPG medium. (**a**) and (**b**) Representative traces of the performed measurements of the rate of oxygen uptake by intact yeast cells in the absence (AOX not expressed) and in the presence (AOX expressed) of galactose. (**c**) Changes in *S. cerevisiae* cell respiration in the presence or the absence of AOX expression and after addition of inhibitors of AOX (BHAM) and MRC complex IV (KCN). The applied concentrations of inhibitors were as follows: 1 mM KCN and 3 mM BHAM. ** *p* < 0.01; *** *p* < 0.001; n/s not statistically significant. (**d**) Detection of *M. inceptum* AOX expression and maintenance in *S. cerevisiae* mitochondria in the presence of galactose. Mitochondria were isolated from cells cultured in the absence of galactose (control) and in its presence, after 2 and 24 h. The red arrows indicate bands corresponding to *M. inceptum* AOX

The performed bioinformatics analysis indicated that the predicted *M. inceptum* AOX displayed a set of properties expected for animal AOX [32, 33]. The predicted protein could be imported into the inner mitochondrial membrane, could form the postulated tertiary structure, and contained the ferritin-like domain and a characteristic motif at the C-terminus defined as a marker of animal AOX (electronic supplementary material, Figure S1 and S2). To functionally assess *M. inceptum* AOX, the predicted protein was expressed in *S. cerevisiae* cells for 24 h under galactose control. Then cell respiration, (i.e., the rate of oxygen uptake) was estimated, and the effects of known inhibitors of AOX (BHAM) and the cytochrome c oxidase (MRC complex IV) representing the MRC cytochrome pathway (KCN) were determined. In the absence of *M. inceptum* AOX expression (Fig. 1A), yeast cell respiration was inhibited by 1 mM KCN but not by 3 mM BHAM, independently of the order of their additions. In the presence of *M. inceptum* AOX expression (Fig. 1B), the inhibitory effect of KCN was distinctly diminished and the resulting KCN-resistant respiration was sensitive to BHAM. Importantly, BHAM-sensitive respiration was also observed before KCN addition. As shown in Fig. 1C, respiration of *S. cerevisiae* cells not expressing *M. inceptum* AOX was sensitive to KCN but not to BHAM, whereas respiration of yeast cells expressing *M. inceptum* AOX was sensitive to BHAM and only partially to KCN. Moreover, basal respiration of *S. cerevisiae* cells expressing *M. inceptum* AOX was approximately two times higher than that of cells not expressing *M. inceptum* AOX but the difference was eliminated by addition of BHAM. Accordingly, as shown in Fig. 1D, only mitochondria isolated from yeast cells cultured in the presence of galactose, triggering AOX expression, contained distinct amounts of a protein of molecular weight close to 40 kD, which corresponds to the molecular weight of heterologously expressed animal AOX proteins [42].

### The presence of BHAM during tun formation but not during tun rehydration affects *M. inceptum* recovery to full activity

**Fig. 2:**
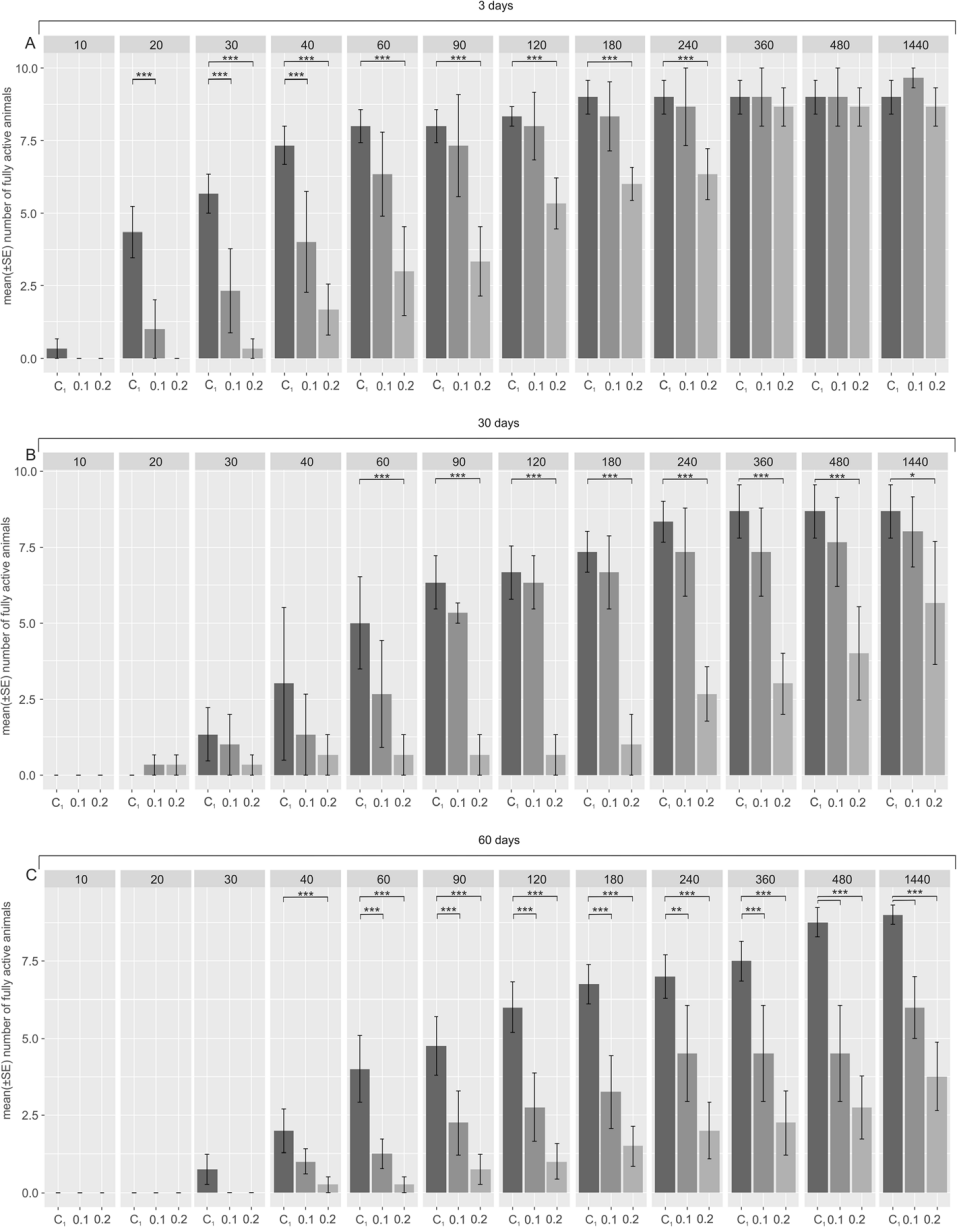
The effect of BHAM presence during *M. inceptum* tun formation on return to full activity. (**a**), (**b**) and (**c**) Tun duration for 3, 30 and 60 days, respectively. Full activity was defined as coordinated movements of animal body and legs (crawling). The time windows of observations are indicated in minutes following the onset of rehydration. C_1_, control 1 (0.3% methanol in the culture medium); 0.1 and 0.2, two different BHAM concentrations (i.e., 0.1 and 0.2 mM BHAM, respectively). * *p* < 0.05; ** *p* < 0.01; *** *p* < 0.001 (see also electronic supplementary material, Table S2)

As expected (e.g., [15]), for the studied duration of the tun stage, (i.e., 3, 30 and 60 days; Fig. 2A – C, respectively) tardigrade revival, corresponding to return to full activity, was delayed as the tun stage duration extended.

The presence of 0.1 or 0.2 mM BHAM during tun formation additively affected the return to full activity in a manner that was dependent on BHAM concentration. Since the *t*-test for the factor “experimental groups” (i.e. control 1, 1 mM BHAM and 0.2 mM BHAM) was statistically significant, the Tukey post-hoc test was developed to test differences between (I) control 1 vs. 0.1 mM BHAM and (II) control 1 vs. 0.2 mM BHAM for different duration of the tun stage (Fig. 2 and electronic supplementary material, Table S2). After the tun stage lasting 3 days (Fig. 2A) statistically significant delay was observed in return to full activity, especially for the higher BHAM concentration, although the final return to full activity was comparable to that of the control group. However, the final return to full activity was distinctly decreased for the tun stage formed in the presence of BHAM and lasting 30 and 60 days. In the case of the former (30 days) a statistically significant decrease was observed only for 0.2 mM BHAM (Fig. 2B), whereas in the case of the latter (60 days), a statistically significant decrease was observed for both BHAM concentrations, and the decrease was more pronounced for 0.2 mM BHAM (Fig. 2C). Thus, the duration of the tun stage as well as the presence of BHAM during tun formation had statistically significant additive effects on return to full activity, which also depended on the AOX inhibitor concentration.

**Fig. 3:**
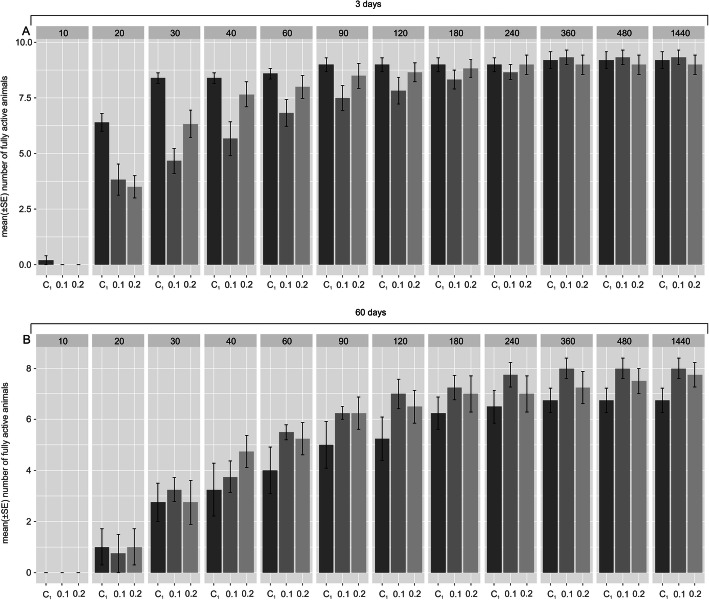
The effect of BHAM presence during *M. inceptum* tun rehydration on return to full activity. (**a**) and (**b**) Tun duration for 3 and 60 days, respectively. Full activity was defined as coordinated movements of animal body and legs (crawling). The time windows of observations are indicated in minutes following the onset of rehydration. C_1_, control 1 (0.3% methanol in the culture medium); 0.1 and 0.2, two different BHAM concentrations (i.e., 0.1 and 0.2 mM BHAM, respectively). The differences observed for the mentioned experimental groups were not statistically significant (see also electronic supplementary material, Table S2)

When added during rehydration, 0.1 mM or 0.2 mM BHAM did not influence return to full activity in a statistically significant way after the tun stage of any duration (Fig. 3 and electronic supplementary material, Table S2). Thus, the presence of BHAM during tun rehydration appeared to not affect animals’ return to full activity.

### BHAM and MitoTEMPO applied during tun formation have different effects on *M. inceptum* recovery to full activity

**Fig. 4:**
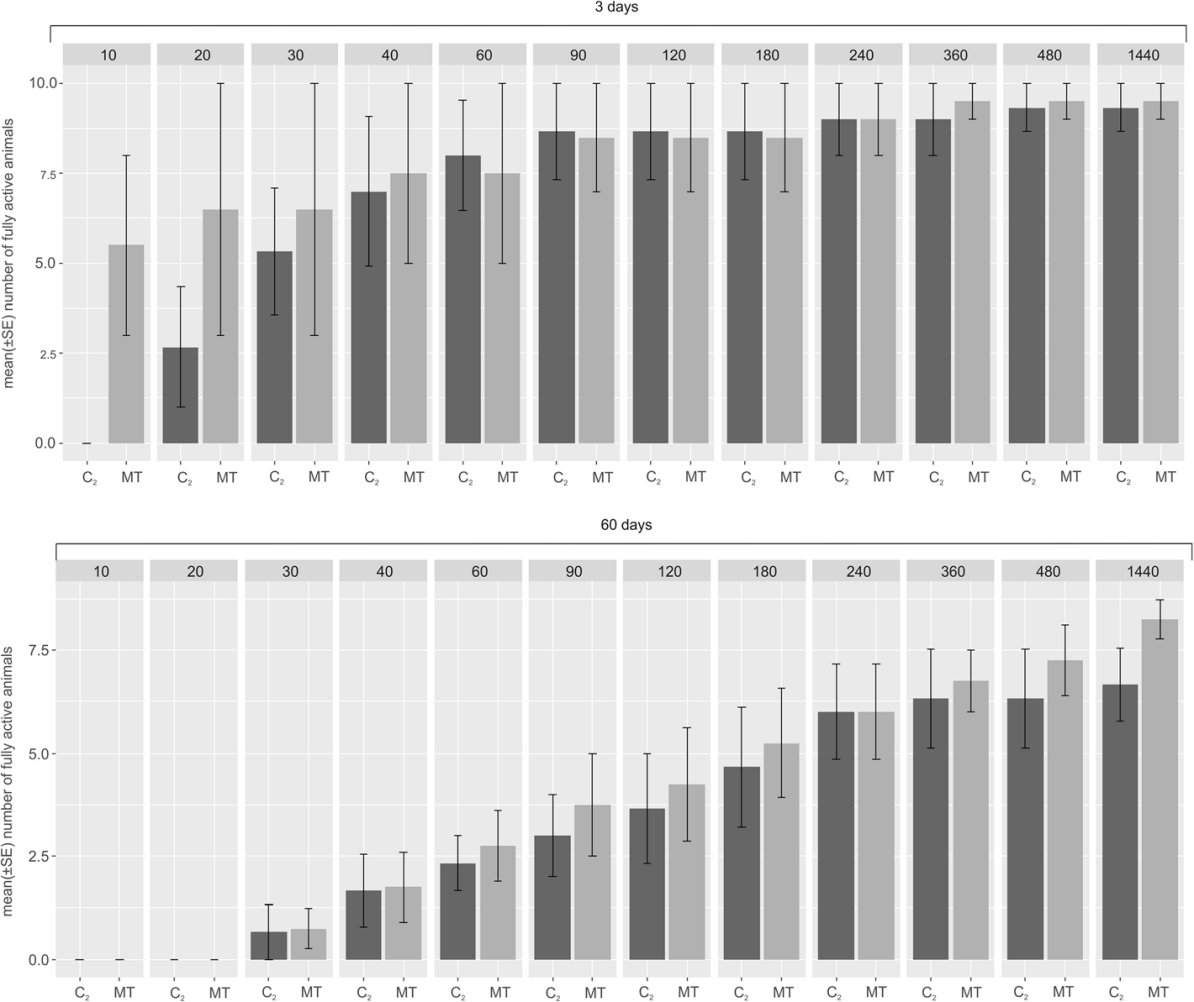
The effect of MitoTEMPO presence during *M. inceptum* tun formation on return to full activity. (**a**) and (**b**) Tun duration for 3 and 60 days, respectively. Full activity was defined as coordinated movements of animal body and legs (crawling). The time windows of observations are indicated in minutes following the onset of rehydration. C_2_, control (the culture medium); MT, 0.01 mM MitoTEMPO. The differences observed for the mentioned experimental groups were not statistically significant (see also electronic supplementary material, Table S3)

MitoTEMPO is a well-known mitochondria-specific superoxide scavenger. Since this activity is also suggested for hydroxamic acids, including BHAM [43, 44], we checked the effect of MitoTEMPO presence during tun formation on animals’ return to full activity. For the studied duration of the tun stage (3 and 60 days), the presence of MitoTEMPO during tun formation did not affect the return to full activity in a statistically significant way (Fig. 4 and electronic supplementary materials, Table S3).

**Fig. 5:**
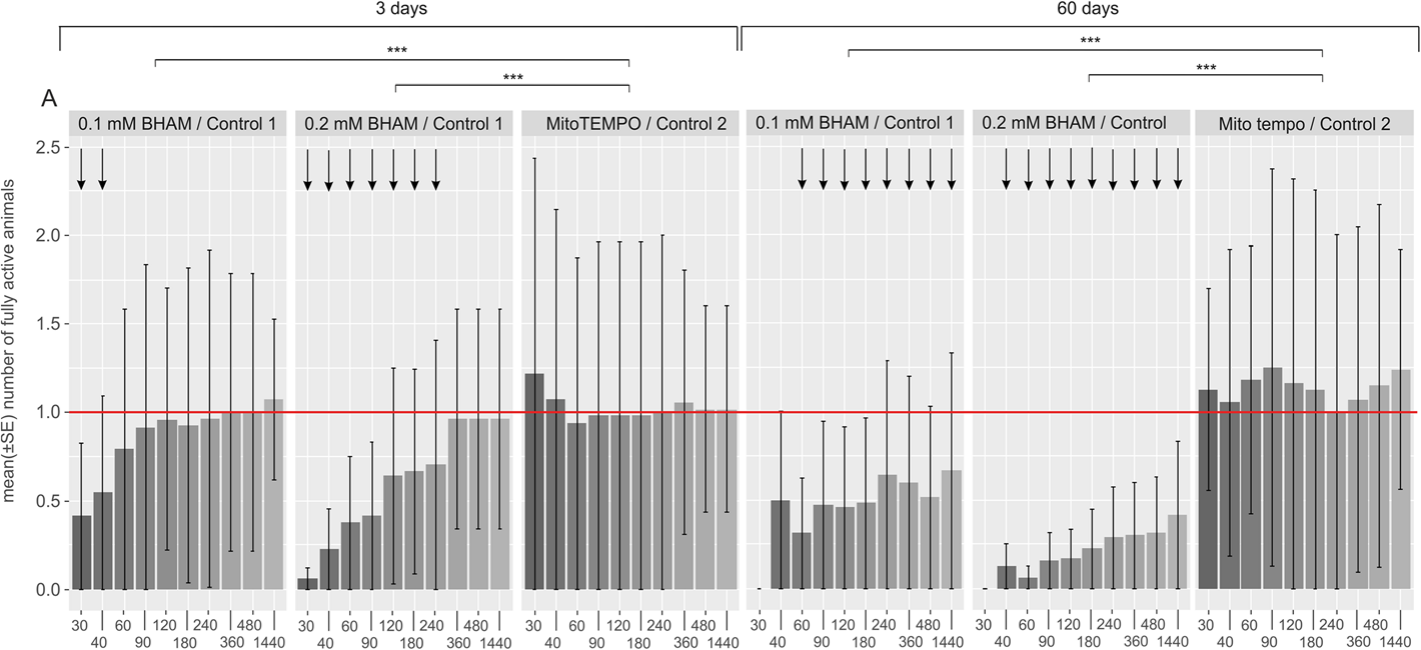
Comparison of the effect of the presence of BHAM and MitoTEMPO during *M. inceptum* tun formation on return to full activity. For the comparison, ratios between fully active animals treated with BHAM (0.1 or 0.2 mM) or MitoTEMPO (0.01 mM) and proper control animals were calculated and analysed by application of the developed Linear Mixed Models (see electronic supplementary material, Table S4). The analysis was performed for different duration of the tun stage. The time windows of observations are indicated in minutes following the onset of rehydration. Full activity was defined as coordinated movements of animal body and legs (crawling). control 1, 0.3% methanol in the culture medium; control 2, the culture medium. *** *p* < 0.001 (see also Tab.  S4). Arrows indicate statistically significant differences between the experimental groups, shown also in Fig. 2

Nevertheless, comparison of effects imposed by the presence of MitoTEMPO or BHAM during tun formation (Fig. 5 and electronic supplementary material, Table S4) indicated that animals forming tuns in the presence of MitoTEMPO displayed faster return to full activity than animals forming tuns in the presence of BHAM. Furthermore, the BHAM effect was distinctly dependent on its concentration and the tun stage duration. Thus, the effects of BHAM and MitoTEMPO imposed during tun formation and exerted on tardigrade recovery to full activity were different. That observation suggested that the effect of BHAM is based on different mechanisms than that of MitoTEMPO. It should be also mentioned that the average number of *M. inceptum* tuns with the expected appearance [16] was not changed by their formation in the presence of BHAM and MitoTEMPO (electronic supplementary material, Fig. S4).

## Discussion

Here we provide data on functionality of the tardigrade *M. inceptum* AOX and its possible contribution to the animal anhydrobiosis. The presence of functional AOX in the tardigrade mitochondria was confirmed bioinformatically (electronic supplementary material, Fig. S1 and S2) and experimentally by heterologous expression of the protein in *S. cerevisiae* cells. As shown in Fig. 1, *M. inceptum* AOX conferred KCN-resistant and AOX inhibitor-sensitive respiration to *S. cerevisiae* cells. The analogous observation was reported for the pacific oyster *Crassostrea gigas* AOX heterologously expressed in yeast mitochondria [42]. Similarly, heterologous expression of the tunicate *Ciona intestinalis* AOX enables in vivo KCN tolerance in *Drosophila melanogaster* [45] and mice [46, 47], observed also for the isolated mitochondria [45–47]. However, in mouse and *D. melanogaster* mitochondria, the enzyme became enzymatically active only when the MRC cytochrome pathway was inhibited beyond coenzyme Q [31, 47–49], whereas in *S. cerevisiae* mitochondria the activity was observed without the cytochrome pathway inhibition [42]. The data available for *C. gigas* AOX studied in the oyster isolated mitochondria do not allow conclusions about the underlying mechanisms [50]. Nevertheless, the data on *C. gigas* AOX expressed in *S. cerevisiae* mitochondria [42] correspond to *M. inceptum* AOX based respiration of *S. cerevisiae* cells (Fig. 1). The difference in regulation of animal AOX in *S. cerevisiae* and animal mitochondria can be explained by differences in organization of the animal and *S. cerevisiae* MRC, including complex I which precedes coenzyme Q in animal MRC but is absent in *S. cerevisiae* mitochondria [51]. Accordingly, it has recently been shown that in the absence of the MRC cytochrome pathway inhibitors, respiratory activity of complex II, but not complex I, both providing electrons to coenzyme Q, is a pre-requisite for the engagement of AOX activity [31].

The engagement could result in coenzyme Q oxidation and consequently diminution of Reactive Oxygen Species (ROS) generation providing damages to DNA, proteins and phospholipids [52–54]. Thus, the enzyme may be considered an important element of protection against ROS-mediated oxidative stress indicated for anhydrobiosis. Oxidative stress can occur during tun formation (dehydration) and tun rehydration [2, 15, 55] due to different mechanisms. During dehydration oxidative stress may result from gradual elimination of water and deprivation of appropriate oxygen availability [2], whereas during rehydration, it may be triggered by reoxygenation [56] and reverse electron transport [28, 57]. Moreover, it is suggested that the decline in revival with increasing time in the tun stage may be caused by oxidative stress based on non-enzymatic and/or enzymatic reactions [2, 15, 58]. Accordingly, in *M. inceptum*, the amount of oxidative DNA damage increases with duration of the tun stage [59]. Thus, if the tardigrade AOX contributes to protection against oxidative stress, the protection should be distinctly impaired in the presence of AOX inhibitor (e.g., BHAM).

The impact of BHAM presence during tun formation on return to full activity by anhydrobiotic *M. inceptum* specimens appears to suggest that AOX has an important role during dehydration (Fig. 2) but not during rehydration (Fig. 3). Moreover, tun formation in the presence of BHAM enhanced the well-known effect of the tun stage duration on anhydrobiotic tardigrade recovery to full activity [12, 15, 60]. We observed that the duration of the tun stage as well as the presence of BHAM during tun formation had additive effects on animals’ return to full activity in a BHAM concentration-dependent manner. Therefore, it may be assumed that AOX contributes to anti-oxidative protection systems underlying dehydration tolerance. Thus, AOX inhibition could impair anti-oxidative protection and, consequently, tardigrade revival.

However, the question arises whether the AOX contribution is more pronounced during dehydration or during the tun stage itself. Taking into account that the presence of BHAM, independently of the applied concentration, did not impair the average number of formed tuns (electronic supplementary material, Fig. S4), one could assume that the activity of AOX is important during the tun stage. Consequently, AOX impairment caused by its inhibitor would result in additional impairment of revival after the tun stage, illustrated by the delay in return to full activity and/or the decreased final return to full activity. Since we observed such an effect of BHAM presence, with revival being dependent on the inhibitor concentration and the tun stage duration (Fig. 2), we assume that AOX contributes to *M. inceptum* tun revival and that its contribution increases with the tun stage duration. As mentioned above, the duration of the tun stage correlates with the possibility of oxidative damage occurrence [59]. One could ask how it is possible for AOX to be active in the nearly complete absence of water. As summarized in [18], water replacement, vitrification (i.e., biological glass formation) and molecular shielding are putative mechanisms enabling dehydration survival. They probably overlap and probably synergistically promote dehydration tolerance that might be based on activity of some proteins. Accordingly, it is suggested that in the presence of some polar molecules including glycerol or trehalose, cells are able to simulate the types of molecular interactions (like hydrogen bonding) that would occur in water [61].

The apparent role of AOX in long-term anhydrobiosis can be explained by its commonly mentioned anti-oxidative effect. BHAM itself may also display such an activity [43, 44], but our data indicate that the effect observed for BHAM (Fig. 2) differs distinctly from that imposed by MitoTEMPO, a well-known mitochondria-specific superoxide scavenger (Figs. 4 and 5). This, in turn, implies that AOX contribution is much more complex. On the one hand, AOX may ensure efficient ATP synthesis under constrained activity of the MRC cytochrome pathway. The possibility of ATP synthesis appears to be pertinent for successful aestivation [62], an important animal survival strategy that, like anhydrobiosis, requires reversible transitions to and from hypometabolic states [63]. On the other hand, AOX may contribute to the control of ROS generation by MRC modulation, also in invertebrate cells [46, 64]. Thus, the obtained results confirm the importance of AOX research in explaining the role of mitochondria in successful anhydrobiosis that may have application in medicine, biotechnology and even space travels.

## Supplementary Information

**Additional file 1: Table S1.** Manufacturers of chemicals applied in the studies. **Table S2.** Results of Factorial ANOVA. **Table S3.** Results of Factorial ANOVA. **Table S4.** Results for Linear Mixed Models developed to compare effects of BHAM and MitoTEMPO on animals’ return to full activity after the tun stage of different duration. **Figure S1.** Bioinformatics analysis of the putative *M. inceptum* AOX amino acid sequence. **Figure S2.** Bioinformatic identification of *M. inceptum* AOX. **Figure S3.** Integration of the AOX gene into the yeast genome. **Figure S4.** The average numbers of tuns formed by *M. inceptum* specimens in the absence and in the presence of BHAM and MitoTEMPO.

## Data Availability

They will be deposited on an open-access platform.
