# Supplementary material for: Mitochondrial alternative oxidase contributes to successful tardigrade anhydrobiosis
Source: Front Zool. 2021 Apr 1;18:15. doi: 10.1186/s12983-021-00400-5 (PMC8015188; doi:10.1186/s12983-021-00400-5)
Supplement: Supplementary file 1 — Additional file 1: Table S1. Manufacturers of chemicals applied in the studies. Table S2. Results of Factorial ANOVA. Table S3. Results of Factorial ANOVA. Table S4. Results for Linear Mixed Models developed to compare effects of BHAM and MitoTEMPO on animals’ return to full activity after the tun stage of different duration. Figure S1. Bioinformatics analysis of the putative M. inceptum AOX amino acid sequence. Figure S2. Bioinformatic identification of M. inceptum AOX. Figure S3. Integration of the AOX gene into the yeast genome. Figure S4. The average numbers of tuns formed by M. inceptum specimens in the absence and in the presence of BHAM and MitoTEMPO. [file 12983_2021_400_MOESM1_ESM.docx]

**Electronic supplementary material**

**TABLES:**

**TABLE S1**

| PCR and PCR product purification required for *M. inceptum* AOX expression in yeast mitochondria | New England Biolabs: Phusion® High-Fidelity PCR Kit (#E0553S) and Monarch® PCR & DNA Cleanup Kit (#T1030S) |
| --- | --- |
| *Saccharomyces cerevisiae* cultures | BD Biosciences: Difico Yeast Extract (#212750), Bacto Peptone (#211677) and Difico Yeast Nitrogen Base without Amino Acids (#231810) |
|  | Sigma-Aldrich: glycerol (#G2025), galactose (#G0625) and Yeast Synthetic Drop-out Medium Supplements without Uracil (#Y1501) |
|  | POCH S.A.: D-glucose (#200-075-1) |
| functional analysis of AOX and anhydrobiosis protocol | Sigma-Aldrich: BHAM (benzohydroxamic acid; #412260), MitoTEMPO ((2-(2,2,6,6-Tetramethylpiperidin-1-oxyl-4-ylamino)-2-oxoethyl)triphenylphosphonium chloride; #SML0737) and KCN (potassium cyanide; #60178) |

**Table S1. Manufacturers of chemicals applied in the studies.**

**TABLE S2**

| model | Factorial ANOVA model | | | | Factors in model | | |
| --- | --- | --- | --- | --- | --- | --- | --- |
|  | ***F-test*_(DF)_** | ***p*** | **R^2^** | **Factor** | | ***t*** | ***p*** |
| Dehydration  3 day tuns | 28.21_(2.105)_ | <0.001 | 0.33 | Time window | | 6.15 | < 0.001 |
|  |  |  |  | Experimental group | | -4.30 | <0.001 |
| Dehydration 30 day tuns | 39.78_(2.105)_ | <0.001 | 0.42 | Time window | | 6.75 | <0.001 |
|  |  |  |  | Experimental group | | -5.82 | <0.001 |
| Dehydration 60 day tuns | 67.92_(2.141)_ | <0.001 | 0.48 | Time window | | 8.60 | <0.001 |
|  |  |  |  | Experimental group | | -7.85 | <0.001 |
| Rehydration  3 day tuns | 18.07_(2.201)_ | <0.001 | 0.14 | Time window | | 5.85 | <0.001 |
|  |  |  |  | Experimental group | | -1.34 | 0.17 |
| Rehydration  60 day tuns | 23.37_(2.141)_ | <0.001 | 0.23 | Time window | | 6.67 | <0.001 |
|  |  |  |  | Experimental group | | 1.48 | 0.14 |

**Table S2.** **Results of Factorial ANOVA.** For each experiment the models included two factors:(I) 12 time windows (10, 20, 30, 40, 60, 90, 120, 180, 240, 360, 480 and 1440 min) and (II) three experimental groups (control, 1 mM BHAM and 0.2 mM BHAM). *F-test*, variation between sample means/variation within the samples; DF, mean degree of freedom used to variance calculations; *p*, level of α significance; R^2^, variance explained; *t,* statistics for factor inside ANOVA model (see also figure 2-3).

**TABLE S3**

| model | Factorial ANOVA model | | | | Factors in model | | |
| --- | --- | --- | --- | --- | --- | --- | --- |
|  | ***F-test*_(DF)_** | ***p*** | **R^2^** | **Factor** | | ***t*** | ***p*** |
| Dehydration  3 day tuns | 4.55_(2.57)_ | 0.014 | 0.10 | Time window | | 2.78 | 0.007 |
|  |  |  |  | Experimental group | | 1.16 | 0.250 |
| Dehydration 60 day tuns | 2.26_(2.81)_ | <0.001 | 0.36 | Time window | | 7.05 | <0.001 |
|  |  |  |  | Experimental group | | 0.84 | 0.399 |

**Table S3**. **Results of Factorial ANOVA.** For each experiment the models included two factors:(I) 12 time windows (10, 20, 30, 40, 60, 90, 120, 180, 240, 360, 480 and 1440 min) and (II) two experimental groups (control, 0.01 mM MitoTEMPO). *F-test*, variation between sample means/variation within the samples; DF, mean degree of freedom used to variance calculations; *p*, level of α significance; R^2^, variance explained; *t,* statistics for factor inside ANOVA model (see also figure 4).

**TABLE S4**

| Group |  | Linear Mixed Model | | Variable inside the Linear Mixed Model | | | |
| --- | --- | --- | --- | --- | --- | --- | --- |
|  |  | ***F*** | ***p*** | **variables** | ***t* (for fixed variable)** | **Chi.sq (for random variable)** | ***p*** |
| Dehydration  3 day tuns | 0.1 mM BHAM / 10 µM MT | 9.01 | < 0.001 | Experimental group | -3.00 | - | 0.004 |
|  |  |  |  | Time window | - | 17.86 | <0.001 |
| Dehydration  3 day tuns | 0.2 mM BHAM / 10 µM MT | 9.03 | 0.004 | Experimental group | -2.73 | - | <0.006 |
|  |  |  |  | Time window | - | 10.7 | 0.003 |
| Dehydration  60 day tuns | 0.1 mM BHAM / 10 µM MT | 7.00 | <0.001 | Experimental group | 3.02 |  | <0.001 |
|  |  |  |  | Time window | - | 12.7 | <0.001 |
| Dehydration 60 day tuns | 0.2 mM BHAM / 10 µM MT | 12.89 | <0.001 | Experimental group | 6.78 | - | <0.001 |
|  |  |  |  | Time window | - | 13.65 | <0.001 |

**Table S4. Results for Linear Mixed Models developed to compare effects of BHAM and MitoTEMPO on animals’ return to full activity after the tun stage of different duration.** Ratios of average numbers of revived animals obtained for BHAM- or MitoTEMPO-treated tardigrades to average numbers of fully active appropriate control animals were analysed, regardless of the time windows**.** The models were performed for different duration of the tun stage (3 and 30 days) and concentrations of BHAM (0.1 mM and 0.2 mM) or MitoTEMPO (0.01 mM). Concentration was used as a fixed effect and time window as a random effect. *F-test*, variation between sample means / variation within the samples; *p*, level of α significance; *t,* statistics for factor inside Linear Mixed Model (see also figure 5).

**FIGURES**

**Figure S1**

**
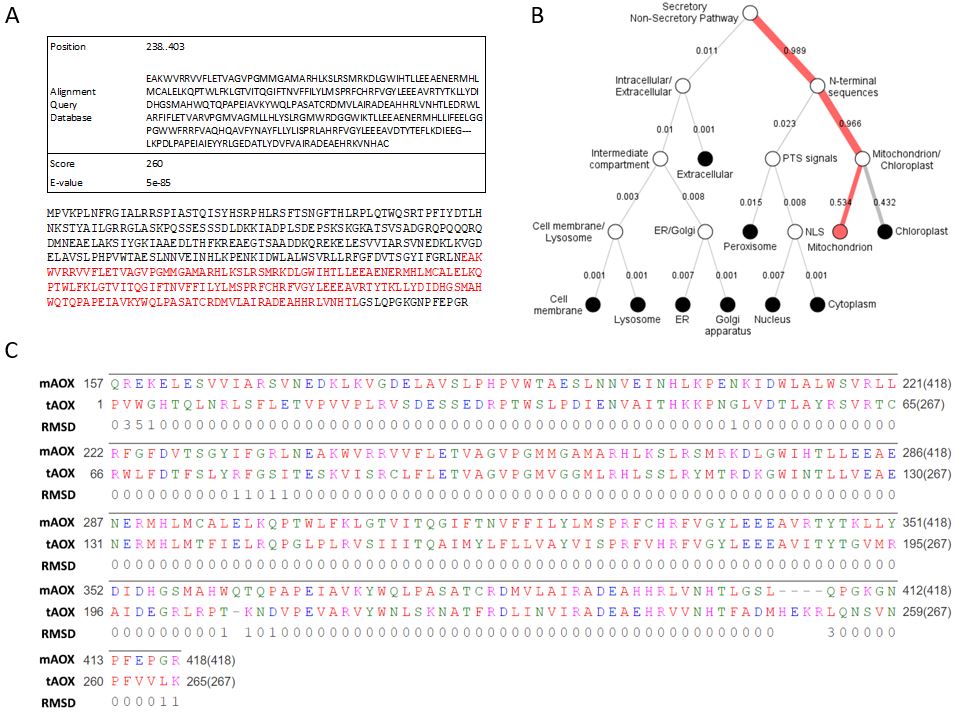
**

**Figure S1. Bioinformatics analysis of the putative *M. inceptum* AOX amino acid sequence.** (A) Identification of structural motives by MotifFinder being a part of I-TASSER (Iterative Threading ASSEmbly Refinement) method. The approach was used to search for motives reported for animal AOX (e.g. McDonald et al., 2009; Pennisi et al., 2016). (B) Cellular localization by DeepLoc-1.0 analysis (www.cbs.dtu.dk/services/DeepLoc/index.php). (C) The root-mean-square deviation of atomic positions (RMSD) indicating average distances between atoms of *M. inceptum* and *Trypanosoma brucei brucei* in their superimposed three-dimensional structures (see also electronic supplementary material, figure S2). The FASTA file of the contig containing the *M. inceptum AOX* gene and the corresponding GFF file containing the genomic location of the gene, transcript, exons and coding DNA sequences) were a kind gift of Dr Felix Bemm (Max Planck Institute for Developmental Biology, Tübingen, Germany).

**Figure S2**

**
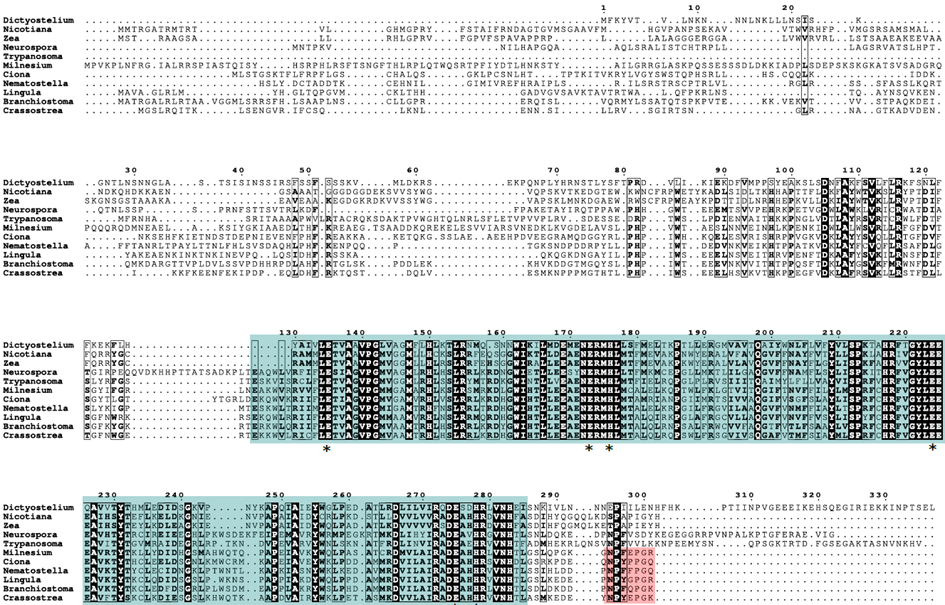
**

**
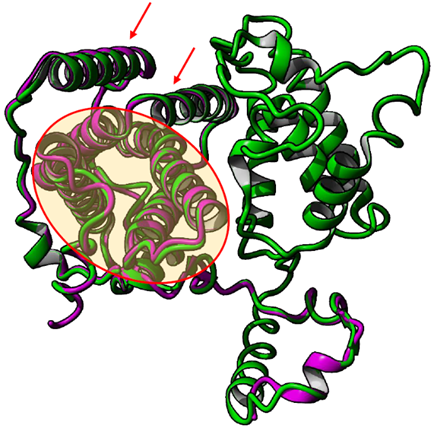
**

**Figure S2. Bioinformatic identification of *M. inceptum* AOX.** (A) A multiple sequence alignment of full-length alternative oxidase (AOX) proteins from selected animal species and representatives of other phylogenetic lineages obtained by Clustal Omega and visualized by ESPript 3.0. The conserved glutamate (E) and histidine (H) residues within the ferritin-like domain (blue box) are marked with stars. The presence of N-P-[YF]-X-P-G-[KQE] motif at C-terminus regarded as diagnostic for animal AOX identification is marked by violet box. Animal species (Opisthokonta) are represented *by Ciona intestinalis* (TIGR genome (Dehal et al., 2002))*, Nematostella vectensis (*NCBI; XM 001635879), *Lingula anatina* (NCBI; XP_013379624.1)*, Branchiostoma floridae* (JGI genome (Putnam et al., 2008)) *and Crassostrea gigas* (NCBI; FJ607013)*.* The other phylogenetic lineages are represented by *Dictyostelium discoideum* ((NCBI; BAB82989), Amoebozoa) *Nicotiana tabacum* and *Zea mays* ((NCBI; AAC60576) and (NCBI; AAC60576), respectively, Archaeplastida, Plants), *Neurospora crassa* ((AAC37481), Opisthokonta, Fungi) and *Trypanosoma brucei brucei* ((NCBI; AAB46424), Excavata). The species taxonomy follows Keeling et al., 2005. (B) Comparison of 3D structure resolved for *T. brucei* AOX (3VVA; Shiba et al., 2013) and predicted for *M. inceptum* AOX by application of I-TASSER. These models were superimposed by application of RaptorX Structure Alignment Server (Wang et al., 2013) and the predicted solution was visualized using YASARA ([www.yasara.org](http://www.yasara.org)). Red frame and arrows indicate four-α-helix bundle of the catalytic core and two additional α-helices anchoring the protein to the membrane, respectively. purple, *T. brucei* AOX monomer; green, *M. inceptum* protein.

**Figure S3**

**
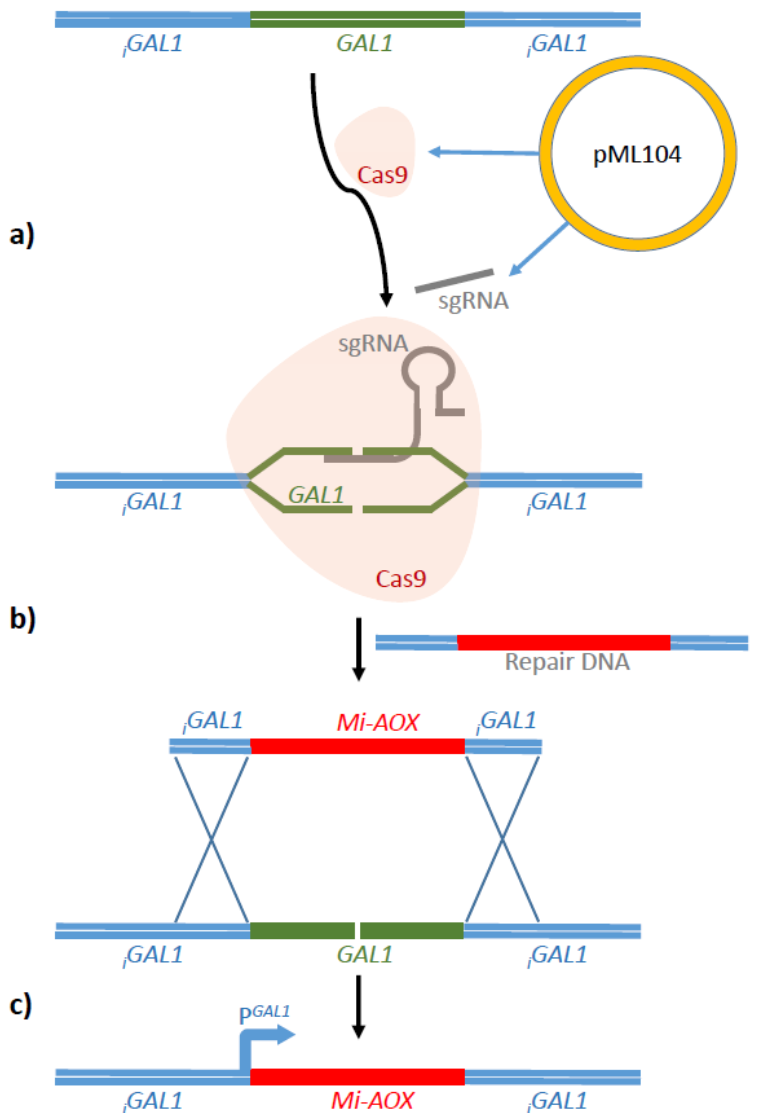
**

**Figure S3. Integration of the *AOX* gene into the yeast genome.** The CRISPR/Cas9 single-guide RNA (sgRNA) targeting *GAL1* was designed to minimize off-targets by using CRISPOR online tool (http://crispor.tefor.net/). Two oligonucleotides: forward primer (5′-GTTTTAGAGCTAGAAATAGCAAGTTAAAATAAGGC-3′) and reverse primer (5′-TTGGACGGTTCTTATGTCACGATCATTTATCTTTCACTGC-3′) carrying the 20mer guide sequence of *GAL1* were used to amplify pML104 plasmid using the Q5 site-directed mutagenesis protocol (New England Biolabs). The resulting pML104-GAL1 was introduced into competent *Escherichia coli* (C2987 strain) cells by heat shock transformation. The plasmid DNA was extracted from the colonies and sequenced by T3 primer. The full-length AOX codon-optimized open reading frame with flanking *GAL1* intergenic sequences was synthesized by Biomatik (Ontario, Canada). The synthetic sequences were received in pBluescript II SK(+) cloning vector which served as a template for repair DNA amplification by PCR (see electronic supplementary materials, Table S1). For the amplification, 20 ng of template vector was mixed with 0.5 µM forward primer (5’- ACGAATCAAATTAACAACCATAGGA-3′) and reverse primer (5’- ATGTCAAGAATAGGTATCCAAAACG-3′), 200 µM dNTPs, 1 x concentrated Phusion HF buffer and 1 U Phusion DNA Polymerase (New England Biolabs). The PCR reaction was performed as follows: an initial denaturation step at 98 ^º^C for 30 s, followed by 30 cycles of denaturation at 98 ^º^C for 8 s, annealing at 55 ^º^C for 10 s, extension at 72 ^º^C for 40 s; and a final extension step at 72 ^º^C for 10 min. The presence of the PCR products was confirmed by gel electrophoresis and the products were then purified by Monarch® PCR & DNA Cleanup Kit (New England Biolabs). The wild-type *S. cerevisiae* BY4741 strain (*MAT*a, *his3*Δ, *leu2*Δ, *met15*Δ, *ura3*Δ) from EUROSCARF were grown in YPD medium containing fermentable carbon source (1% yeast extract, 2% peptone, 2% glucose) to OD_550_ = 4. The pML104-GAL1 plasmid and repair DNA were co-transformed into the yeast cells by electroporation under Gene Pulser Xcell (Bio-Rad) conditions: 25 µF, 200 Ω, 1.5 kV and 0.2 cm cuvette. Then the yeast cells were selected on solid synthetic dextrose (SD) medium containing 0.67% yeast nitrogen base without amino acids, 2% glucose and 0.12% of drop out -ura (mixture of all amino acids and purines without uracil). Genomic DNA was then extracted from the resulting colonies and the presence of AOX encoding sequence was verified by sequencing. (A) Plasmid pML104 expresses Cas9 endonucleases and designed sgRNA, which allows DNA cleavage in the GAL1 region. (B) The repair DNA containing the Mt-AOX gene and the GAL1 gene flanks (intergenic regions - iGAL1) are incorporated into the yeast genome by homologous recombination. (C) Replacement of DNA fragments removes the Cas9 cleavage site and allows expression of the Mt-AOX gene under the control of the GAL1 promoter.

**Figure S4**


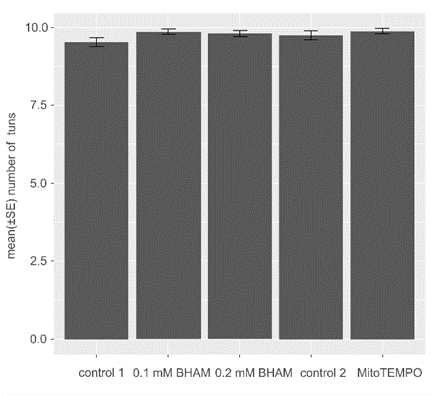


**Figure S4. The average numbers of tuns formed by *M. inceptum* specimens in the absence and in the presence of BHAM and MitoTEMPO.** The expected appearance of tuns included compact body shape resulting from contracting the body and withdrawal of the legs into the body cavity accompanied by the body water loss. To obtain the tun stage groups of 10 fully active adult specimens (4-6 replicates) were dehydrated in the absence or in the presence of BHAM (0.1 or 0.2 mM) and MitoTEMPO (0.01 mM). The full activity was defined as coordinated movements of the animal body and legs (crawling). control 1, 0.3% methanol in the culture medium (methanol was used as BHAM solvent); control 2, the culture medium. The differences between the means are not statistically significant (one-way ANOVA: F_4,89_ = 1.65 *p* = 0.167).
